# Increased p53 immunopositivity in anaplastic medulloblastoma and supratentorial PNET is not caused by JC virus

**DOI:** 10.1186/1471-2407-5-19

**Published:** 2005-02-17

**Authors:** Charles G Eberhart, Aneeka Chaudhry, Richard W Daniel, Leila Khaki, Keerti V Shah, Patti E Gravitt

**Affiliations:** 1 Department of Pathology, Johns Hopkins University School of Medicine, Ross Bldg 558, 720 Rutland Ave, Baltimore, MD 21205, USA; 2 Department of Epidemiology, The Johns Hopkins Bloomberg School of Public Health, 615 N. Wolfe St., Room E6535 Baltimore, MD 21205, USA; 3 Department of Molecular Microbiology and Immunology, Johns Hopkins Bloomberg School of Public Health, 615 North Wolfe Street, Baltimore, MD 21205, USA

## Abstract

**Background:**

p53 mutations are relatively uncommon in medulloblastoma, but abnormalities in this cell cycle pathway have been associated with anaplasia and worse clinical outcomes. We correlated p53 protein expression with pathological subtype and clinical outcome in 75 embryonal brain tumors. The presence of JC virus, which results in p53 protein accumulation, was also examined.

**Methods:**

p53 protein levels were evaluated semi-quantitatively in 64 medulloblastomas, 3 atypical teratoid rhabdoid tumors (ATRT), and 8 supratentorial primitive neuroectodermal tumors (sPNET) using immunohistochemistry. JC viral sequences were analyzed in DNA extracted from 33 frozen medulloblastoma and PNET samples using quantitative polymerase chain reaction.

**Results:**

p53 expression was detected in 18% of non-anaplastic medulloblastomas, 45% of anaplastic medulloblastomas, 67% of ATRT, and 88% of sPNET. The increased p53 immunoreactivity in anaplastic medulloblastoma, ATRT, and sPNET was statistically significant. Log rank analysis of clinical outcome revealed significantly shorter survival in patients with p53 immunopositive embryonal tumors. No JC virus was identified in the embryonal brain tumor samples, while an endogenous human retrovirus (ERV-3) was readily detected.

**Conclusion:**

Immunoreactivity for p53 protein is more common in anaplastic medulloblastomas, ATRT and sPNET than in non-anaplastic tumors, and is associated with worse clinical outcomes. However, JC virus infection is not responsible for increased levels of p53 protein.

## Background

The current World Health Organization classification for tumors of the nervous system includes medulloblastoma, medulloepithelioma, ependymoblastoma, supratentorial primitive neuroectodermal tumor (sPNET) and atypical teratoid/rhabdoid tumor (ATRT) in the category of embryonal brain neoplasms [1]. These tumors are united by their primitive cytological appearance and the ability to differentiate into multiple cell types. However, recent studies indicate that these lesions are genetically, and to some extent clinically, separable. ATRT are defined by the presence of rhabdoid cells, contain *INI1 *mutations, and cause particularly grim clinical outcomes [2]. Medulloblastomas commonly contain isochromosome 17q, but this chromosomal alteration is rarely detected in sPNET or ATRT [3]. Global gene expression profiles also suggest that medulloblastoma, sPNET and ATRT are distinct entities [4].

Little is known about the differences in p53 expression and function among the various embryonal brain tumor subtypes. Initial reports on the p53 tumor suppressor gene suggested it was mutated in 10% or less of medulloblastomas [5-8]. However, Frank and colleagues have recently shown that the p53 pathway is inactivated by mutation of p53, methylation of p14ARF, or deletion of INK4/ARF in 21% of medulloblastomas [9]. In their study, 5 of the 6 medulloblastomas with alterations abrogating p53 function had significant anaplasia. Large cell/anaplastic changes in medulloblastoma are prognostic of significantly worse clinical outcomes [10-12]. Interestingly, p53 protein accumulation, which is often associated with loss of functionality, has been found by some [13-15], but not others [16,17], to predict shorter survival in medulloblastoma patients as well.

Several other lines of evidence also support a role for the p53 pathway in medulloblastomas. First, medulloblastomas sometimes arise in the context of Li Fraumeni syndrome, in which p53 germline mutations predispose patients to a wide range of neoplasms [18]. Second, inactivation of p53 accelerates the formation of medulloblastomas in transgenic mouse models [19]. Finally, inactivation of p53 and Rb simultaneously, either through genetic disruption or overexpression of viral T antigen, results in medulloblastomas in rodents [20-22].

It has been suggested that viral infection of human CNS tissues could promote formation of brain tumors by inhibiting p53 and Rb activity [23]. Some researchers have reported the presence of JC virus or other oncogenic polyomaviruses in human brain tumor specimens, including medulloblastomas [24,25]. Large T antigen expressed by these viruses binds and inactivates p53 [26]. This process results in the accumulation and immunohistochemical detection of p53 protein. In human neural tissue this is best demonstrated in progressive multifocal leukoencephalopathy, in which JC virus infected oligodendroglial cells are strongly p53 immunopositive [27,28]. It is therefore possible that the accumulation of p53 protein in some human medulloblastomas is caused by viral infection.

In order to confirm the association between p53 immunopositivity, clinical outcome, and embryonal tumor subtype, we stained a tissue array containing representative cores from 80 embryonal brain tumors for p53. We also investigated JC virus infection as a possible mechanism for accumulation of p53 protein by searching for viral sequences using a highly sensitive quantitative real time polymerase chain reaction (PCR) assay. We found an association between p53 immunoreactivity, clinical outcome, and tumor subtype, but did not detect JC virus in medulloblastoma or supratentorial PNET.

## Methods

### Clinical material

Medulloblastomas and other embryonal brain tumors diagnosed at the Johns Hopkins University Department of Pathology were identified through review of departmental records. Classic, desmoplastic/nodular and large cell/anaplastic medulloblastomas were classified using World Health Organization guidelines [1]. Nuclear size, cell morphology and the frequency of mitosis and apoptosis were used as previously described to grade anaplasia [11]. 80 Tumors from 78 patients were used to create a tissue array as previously described [29]. Patients ranged from 8 months to 55 years of age, with a median age of 9 years. Microscopic examination of the array confirmed that the appearance of tumor tissue cores corresponded to donor blocks. Frozen tumor tissue obtained from medulloblastomas resected at the Johns Hopkins Hospital was snap-frozen in liquid nitrogen and stored at minus 80°C prior to nucleic acid extraction. DNA was extracted using Trizol and further purified using a DNeasy column (Qiagen, Valencia, CA) according to the manufacturer's instructions. This study was approved by the Johns Hopkins University Institutional Review Board.

### Immunohistochemistry

The tissue array was sectioned at four microns, deparaffinized, and subjected to antigen retrieval by steaming (20 minutes at 80°C). Slides were then incubated at room temperature for 45 minutes with monoclonal antibody directed towards p53 (1:2000, clone DO-7, DAKO, Carpinteria, CA). Primary antibody was detected using the avidin-biotin complex (ABC) method with diaminobenzadine serving as the chromagen. We semiquantitatively graded staining intensity as negative, weak, or strong. Carcinomas with mutations leading to p53 stabilization were used as positive controls. No staining was seen in the absence of primary antibody (negative control).

### Detection of virus by real time PCR

JC virus sequences were amplified from DNA using the forward primer PEP-1 (5'-AGT CTT TAG GGT CTT CTA CC-3') and reverse primer PEP-2 (5'-GCC AAC CTA TGG AAC AG-3') [30]. Additional specificity for detection of JC virus was achieved using the FAM/Black Hole Quencher-1 (FAM/BHQ-1) labeled TaqMan probe (5-/56-FAM/ CCA ACA CTC TAC CCC ACC T /3BHQ_1/-3) [31]. This probe does not cross-react to the closely related human BK polyomavirus, or simian SV40 polyomavirus. Fifty microliter reaction volumes were used, comprised of 1X universal master mix (Applied Biosystems, Foster City, CA), 0.05 μM probe, 0.4 μM PEP-1, 0.4 μM PEP-2, and 16–288 ng tumor DNA. Amplifications were performed using a Biorad ICycler with the following thermal profile: 95°C for 10 minutes, then 50 cycles at 95°C for 15 seconds and 57°C for 1 minute. Quantitation of JC virus in tumors was determined using linear regression with an external standard curve included on each plate containing a 5-fold dilution series of known input JC virus plasmid diluted in a constant background of human placental DNA (70 ng/μl). To normalize for sampling variability, we quantitated the total cell equivalents in each sample by amplifying a human endogenous retrovirus gene (ERV-3) in an equal amount of tumor DNA [32]. Conditions for ERV-3 amplification were: 0.4 μM forward primer (PHP10-F: 5'-CAT GGG AAG CAA GGG AAC TAA TG-3'), 0.4 μM reverse primer (PHP10-R: 5'-CCC AGC GAG CAA TAC AGA ATT T-3'), and 0.25 μM TaqMan probe labeled with FAM and BHQ (PHP-P505/ERV-3 Probe: 5'-/56-FAM/TCT TCC CTC GAA CCT GCA CCA TCA AGT CA/3BHQ_1/-3'). ERV-3 amplification was performed on an ABI 5700 using the following thermal profile: 95°C for 10 minutes, followed by 50 cycles of amplification at 95°C for 15 seconds and 60°C for 30 seconds. DNA extracted from the diploid cell line ATCC CCL 205 diluted in a constant background of 50 ng/μl salmon sperm DNA was used to construct the ERV-3 standard dilution curve.

### Statistical analysis

p53 statistical analyses were performed using GraphPad PRISM4 software (GraphPad Software, San Diego, CA). A two tailed Fishers Exact test was used to compare immunohistochemical staining profiles between groups. Significance of survival differences was assessed using log-rank analysis of Kaplan-Meier curves. The formula 1-(alpha)^1/N ^was used to calculate the fiducial (exact) 95% upper bound of JC virus prevalence.

## Results

### p53 protein levels are increased in anaplastic medulloblastoma, ATRT and sPNET

We used immunohistochemistry to examine p53 protein expression in representative cores from 80 embryonal brain tumors on a tissue array. Cores from 5 of the cases could not be evaluated due to crush artefact, cautery, or low cellularity. Of the remaining 75 tumors, 64 were medulloblastomas; 31 of these medulloblastoma were of the classic subtype, 13 were nodular/desmoplastic, and 20 were large cell/anaplastic. The tissue array used in this study also contained 11 other CNS embryonal tumors, including 8 sPNET and 3 ATRT. Among the sPNET were 3 with the long epithelial surfaces characteristic of medulloepithelioma.

**Figure 1:**
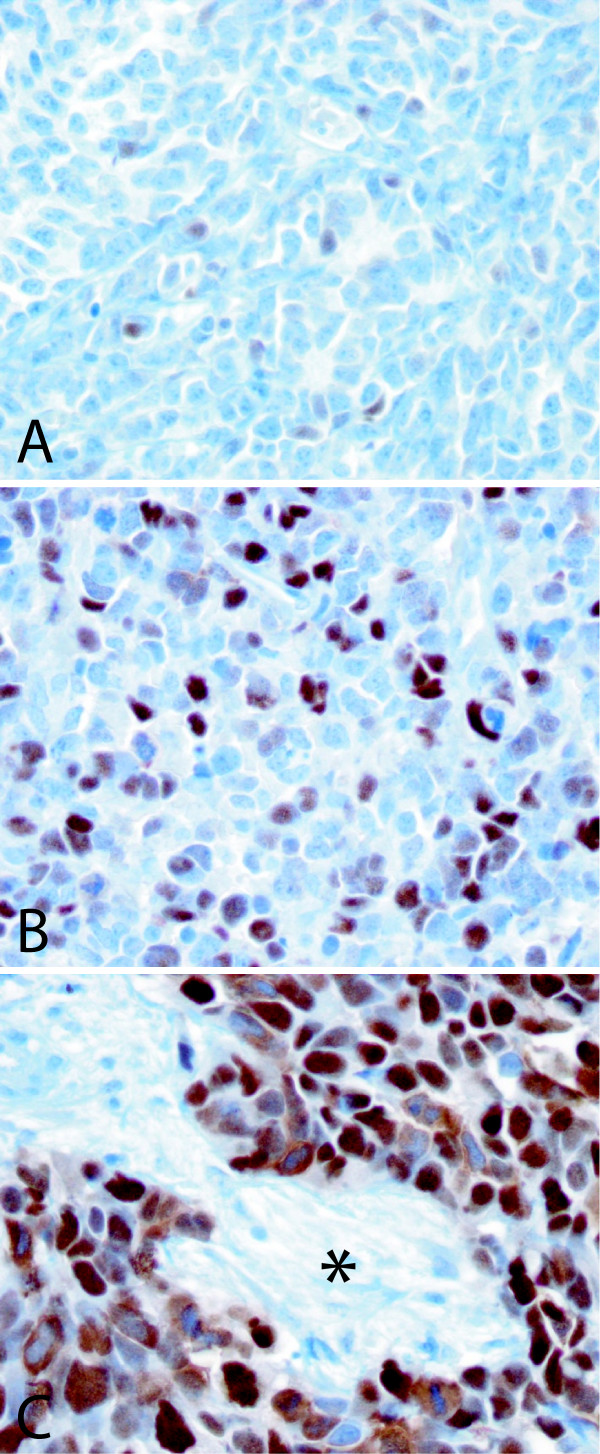
**p53 Immunostaining in CNS embryonal tumors. **Most p53 immunopositivity was relatively faint and present only in a small fraction of the tumor. This nodular medulloblastoma had weak staining in less than 25% of cells (A). Some anaplastic medulloblastomas had strong p53 immunostaining in a larger fraction of cells (B). Most AT/RT and supratentorial PNET contained p53 immunopositive cells, like this lesion metastatic within the CNS in which almost all cells are strongly positive (C). Note the lack pf staining in non-neoplastic stroma (Asterisk).

**Figure 2:**
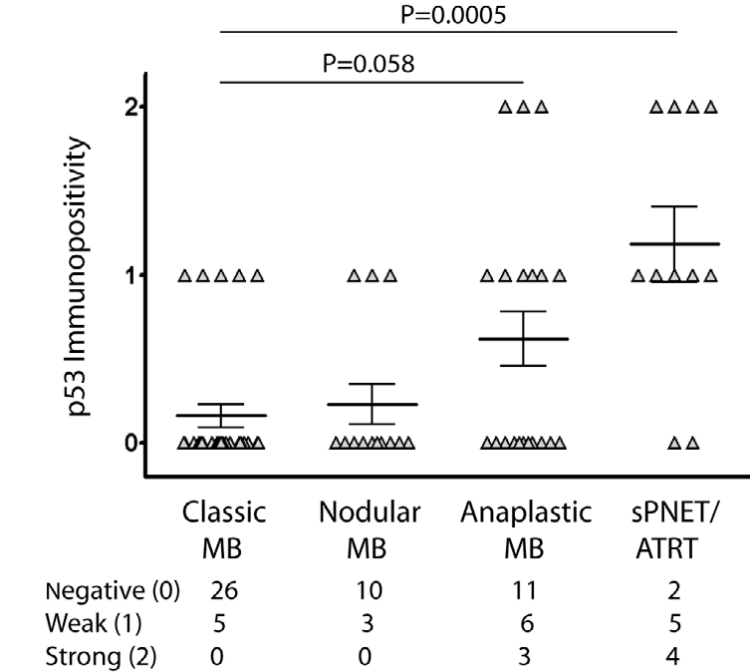
**A statistically significant increase in p53 immunopositivity in AT/RT and sPNET **Most classic and nodular medulloblastoma (MB) contained no p53 immunopositive cells. In contrast, p53 positive cells were present in many anaplastic MB, supratentorial primitive neuroectodermal tumors (sPNET) and atypical teratoid rhabdoid tumors (ATRT). Triangles represent individual tumors with negative (0), weak (1) or strong (2) p53 immunostaining. Bars represent the mean and standard error of the mean. The increase in p53 immunopositivity in the sPNET/ATRT category as compared to classical MB was statistically significant (Mann Whitney test).

Immunoreactivity for p53 was present in a minority (35%) of the 75 embryonal tumors, and a relatively small subset of cells stained in most of the positive cases. In all positive cases the majority of the immunoreactivity was in tumor cell nuclei. An example of a nodular medulloblastoma with weak, scattered p53 immunoreactivity is shown in Figure 1A. A medulloblastomas and a sPNET with strong immunoreactivity are shown in Figure 1B and 1C, respectively. Only 16% (5/31) of classic medulloblastomas and 23% (3 /13) of nodular medulloblastomas were immunopositive for p53, and staining was weak in all of these cases. In contrast, 45% (9/20) of anaplastic medulloblastomas, 67% (2/3) of ATRT, and 88% (7/8) of sPNET were positive for p53. The intensity of staining in the anaplastic medulloblastomas and extracerebellar embryonal tumors was strong in many cases (Figure 2). The increase in p53 immunoreactivity in anaplastic medulloblastomas was statistically significant when compared to non-anaplastic ones, including classic and nodular lesions (P = 0.03, Fisher's Exact test). Other embryonal tumors (sPNET and ATRT) also had a significant increase in p53 expression compared to non-anaplastic medulloblastoma (P = 0.0001, Fisher's exact test). p53 expression was often widespread in the severely anaplastic medulloblastoma, ATRT, and sPNET groups, with 6 of 12 tumors showing immunoreactivity in over 25% of cells, while classic and nodular medulloblastomas always had fewer than 25% immunopositive cells (Table 1). Staining for p53 was distributed evenly in most lesions, rather than being concentrated in focal groups of tumor cells.

### p53 Immunopositivity is significantly associated with worse clinical outcomes

We next examined whether p53 immunopositivity was associated with worse clinical outcomes in embryonal brain tumor patients. Of the 75 tumors from 73 patients scored on the array, survival data was available for 66 individuals. 61 percent of patients were alive at last contact, with follow up times ranging from 3 to 215 months (median 47 months). When survival of the entire embryonal brain tumor cohort was analyzed, 67% (31/46) of patients with p53 immunonegative tumors were alive, as compared to 45% (9/20) of patients with immunopositive tumors. Log rank analysis of Kaplan Meier survival curves confirmed the significance of this difference (P = 0.02). When only the 56 medulloblastoma patients with clinical follow up were analyzed, 71% of individuals with p53 negative tumors survived, compared to 58% of individuals with p53 positive tumors. However, these differences were not significant on log rank analysis (P = 0.25). The 6 patients with intensely immunoreactive tumors (3 anaplastic medulloblastoma, 2 sPNET, 1 ATRT) all died from their disease in less than two years.

### JC virus infection does not account for increased levels of p53 protein

**Figure 3:**
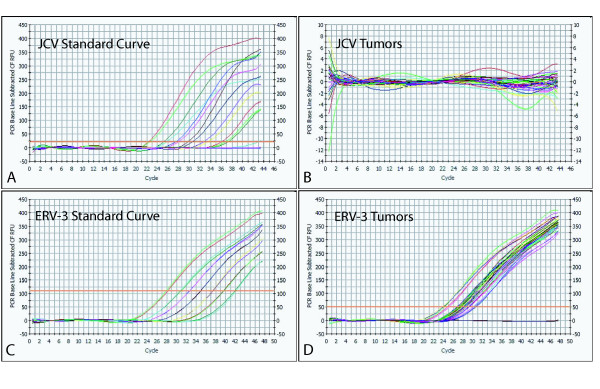
**JC virus sequences are not detected in embryonal brain tumors. **JC virus plasmid DNA is detectable over a wide range of dilutions when added to genomic DNA (A), but was not identified in DNA extracted from a range of embryonal brain tumors (B). In contrast, the ERV-3 endogenous retrovirus is easily detected both using standard dilution curves (C) and in tumor DNA (D).

To address the possibility that p53 accumulation in medulloblastoma and other embryonal brain tumors is due to viral infection, we used quantitative RT PCR to search for viral sequences in tumor DNA. Of the cases used on the tissue array, 19 had material frozen suitable for high-quality DNA extraction. Four of these cases were p53 positive. Also available in our frozen tumor bank was tissue from 14 additional embryonal lesions (4 classic medulloblastoma, 5 anaplastic medulloblastoma, 1 nodular medulloblastoma and 4 sPNET). We did not detect JC virus sequences in any of these 33 tumors. We repeated the analysis on DNA extracted from a different tumor tissue fragment in 5 cases, but these spatially distinct regions also failed to contain viral DNA. ERV-3, an endogenous human retroviral element, served as a control and was easily amplified from tumor DNA samples (Figure 3). ERV-3 copy numbers ranged from ~2 × 10^3 ^- 2 × 10^5 ^per PCR assay (mean 3.4 × 10^4^). Because two copies of ERV-3 are present per human genome, DNA from at least 1000 cells was assayed in each PCR reaction. In contrast, all of the specimens tested were completely negative for JC virus. The quantitative Taqman assay was able to detect a minimum of ~10 copies of JC virus per PCR reaction as determined using a standard dilution curve. Thus the number of JC virus genomes should not exceed 10 per 1,000 tumor cells. Given our 0% detection rate, the 95% confidence limit for the largest value of the 'true' underlying JC virus prevalence is no more than 7.6% (1-(0.05)^1/38^).

## Discussion

To investigate the prognostic potential and pathological role of p53 expression in embryonal brain tumors, we analyzed this protein in 75 medulloblastoma, ATRT and sPNET using immunohistochemistry. Overall, we found significant increases in p53 immunoreactivity in anaplastic medulloblastomas (45% positive) as compared to non-anaplastic ones (18% positive). The percentage of p53 immunopositive medulloblastomas in our study (27%) fell within the previously reported range of 3% to 53% [13,16,17,33,34]. This wide variation in published values is likely due to differences in antibodies used, their dilutions, and antigen retrieval protocols. Cuttoffs for a calling a tumor "positive" also varied among previous investigators, with some scoring only intensely positive lesions. Only 5% of our medulloblastomas fell into this strong staining category, and all of these were anaplastic. Mutation of the p53 gene often results in a stabilized protein of altered functionality that accumulates in the nucleus of tumor cells [35]. Our data are thus consistent with those recently reported by Frank and colleagues, who found that the TP53 pathway was frequently disrupted in large cell/anaplastic medulloblastomas [9].

Interestingly, supratentorial PNET and ATRT were also more commonly p53 immunopositive than non-anaplastic medulloblastoma in our study. While extracerebellar PNET were included in several earlier studies, p53 immunoreactivity was not reported separately for these lesions [14,15]. Ho and colleagues documented p53 mutations in 6 of 14 sPNET but did not examine protein expression [36]. In another relatively large series, only 1 of 12 sPNET contained a mutation in the p53 gene [37]. Finally, Postovsky and colleagues described an unusual p53 mutation in a case report of a sPNET [38]. With regard to rhabdoid lesions, Berrak and colleagues documented faint p53 immunoreactivity in 6 of the 7 ATRT of the CNS they examined [39]. Malignant rhabdoid tumors arising outside the CNS are also commonly p53 immunopositive, and mutations predicted to inactivate p53 function have been documented in some [40]. Thus while the number of sPNET and ATRT we examined was relatively low, our data, combined with earlier reports, suggests that p53 function may be commonly altered in embryonal tumors arising outside the cerebellum.

Clinical outcomes were significantly worse for embryonal brain tumor patients in our study whose lesions were p53 immunopositive. However, cases most commonly positive for p53 (anaplastic medulloblastomas, sPNET, and ATRT) are all more clinically aggressive than non-anaplastic medulloblastomas, making it difficult to infer causality resulting from p53 accumulation. p53 expression did not predict outcome within the group of medulloblastoma patients, although all 3 strongly p53 immunopositive tumors were severely anaplastic and associated with quite short survival. Interestingly, in a recently published report, Ray and colleagues found that p53 immunoreactivity was the only biological marker predictive of poor outcome on both univariate and multivariate analyses in a group of 112 medulloblastoma patients [41].

We also examined the potential role of viral infection as a mechanism for p53 protein stabilization in medulloblastoma. None of the four p53-positive cases from our tissue array with frozen material available contained viral sequences. We also tested nine additional samples of histological subtypes that were more commonly associated with p53 immunopositive staining (anaplastic medulloblastomas and sPNETs) where p53 staining was not performed. None of these tumors were JCV positive. While sample availability precluded complete testing of all confirmed p53 positive tumors for JCV, the available data do not support a causal role for JCV infection in p53 accumulation and development of embryonal brain tumors.

The impact of viruses on medulloblastoma pathogenesis is controversial. The most commonly implicated agents are the polyomavirus family members JC virus, BK virus and SV40 virus. Of these, JC virus, which infects approximately 80% of the pediatric population and can cause CNS disease in immunosuppressed patients, has been most studied. It was shown decades ago that inoculation of JC virus into rodents resulted in formation of medulloblastoma-like cerebellar tumors [20,22,42,43]. Transgenic mice containing JC virus early region sequences also develop medulloblastomas [44]. Del Valle and colleagues isolated JC virus large T antigen sequences from 11 of 23 human medulloblastomas they examined, and suggested inactivation of p53 and Rb by viral T antigen could be important in the pathogenesis of human embryonal brain tumors [24]. A second gene (Agno) from JC virus was later reported to be present in 11 of 16 medulloblastoma samples by the same group [25].

We failed to detect JC virus sequences in the 33 cases examined, using a sensitive and specific technique which should identify as few as 10 viral genomes per PCR reaction. This suggests the p53 immunopositivity we observe is not caused by JC virus infection in the majority of cases. Our data are also not consistent with the hypothesis that ongoing JC virus infection is common in medulloblastoma. Other recent studies have also reported a lack of JC virus DNA in medulloblastomas. Hayashi and colleagues failed to identify JC viral sequences in 13 medulloblastomas [45]. Kim and colleagues similarly failed to identify JC virus in 15 medulloblastomas, 5 sPNET and 2 medulloblastoma cell lines [46]. Rollison and colleagues examined 225 brain tumors, including 20 medulloblastomas, for JC, BK and SV40 viruses at two different laboratories [47]. No tumor tested positive in both laboratories. Finally, Weggen and colleagues failed to detect JC virus sequences in any of 116 medulloblastomas analyzed, although 2 of these cases were SV40 positive [48]. These reports do not rule out the possibility of a "hit and run" process in which virus participates initially in the formation of a lesion and is then lost. They do strongly suggest that ongoing JC virus infection is not common in human medulloblastomas, as was initially suggested. Interestingly, it has recently been shown that the putative SV40 infection of human mesotheliomas can be accounted for by contamination of samples with small amounts of common laboratory plasmids containing regions of the T-antigen gene [49], calling into question the true association of SV40 with human cancers, including brain tumors.

## Conclusion

In summary, we find significantly increased p53 protein levels in anaplastic medulloblastomas, sPNET, and ATRT as compared to classic and nodular medulloblastoma. JC virus was not detected in the 33 tumors examined, suggesting that T-antigen binding does not appear to be an ongoing factor in the pathobiology or p53 protein accumulation of embryonal brain tumors.

## Competing interests

The author(s) declare that they have no competing interests.

## Authors' contributions

Drs. Eberhart, Shah and Gravitt planned the study and wrote the initial manuscript draft. Dr. Eberhart, Ms. Chaudhry and Ms. Khaki collected samples, isolated DNA, and performed immunohistochemical staining. Dr. Gravitt supervised and R Daniel performed the JC virus copy number analysis in tumors. All authors reviewed and commented on the final manuscript.

## Pre-publication history

The pre-publication history for this paper can be accessed here:



## Figures and Tables

**Table 1:** Intensity and extent of p53 immunopositivity in CNS embryonal tumors

Case	Tumor Subtype	p53 Intensity	p53 Positive Cells
1	Classic MB	1	5 to 25%
2	Classic MB	1	5 to 25%
3	Classic MB	1	5 to 25%
4	Classic MB	1	5 to 25%
5	Classic MB	1	5 to 25%
6	Nodular MB	1	5 to 25%
7	Nodular MB	1	5 to 25%
8	Nodular MB	1	5 to 25%
9	Moderately Anaplastic MB	1	5 to 25%
10	Moderately Anaplastic MB	1	5 to 25%
11	Moderately Anaplastic MB	1	5 to 25%
12	Moderately Anaplastic MB	1	5 to 25%
13	Moderately Anaplastic MB	1	5 to 25%
14	Moderately Anaplastic MB	1	5 to 25%
15	Severely Anaplastic MB	2	51 to 75%
16	Severely Anaplastic MB	2	51 to 75%
17	Severely Anaplastic MB	2	26 to 50%
			
18	sPNET	1	5 to 25%
19	sPNET	1	5 to 25%
20	sPNET	1	5 to 25%
21	sPNET	2	5 to 25%
22	sPNET/Medulloepithelioma	1	5 to 25%
23A	sPNET/Medulloepithelioma	2	51 to 75%
23B	sPNET/Medulloepithelioma	2	76 to 100%
24	AT/RT	1	5 to 25%
25	AT/RT	2	26 to 50%

MB – medulloblastoma; sPNET – supratentorial PNET; AT/RT – atypical teratoid/rhabdoid tumor; p53 Intensity (0 – negative, 1 – weak, 2 – strong).

## References

[B1] Kleihues P, Cavenee WK (2000). Tumors of the Nervous System.

[B2] Biegel JA, Fogelgren B, Zhou JY, James CD, Janss AJ, Allen JC, Zagzag D, Raffel C, Rorke LB (2000). Mutations of the INI1 rhabdoid tumor suppressor gene in medulloblastomas and primitive neuroectodermal tumors of the central nervous system. Clin Cancer Res.

[B3] Russo C, Pellarin M, Tingby O, Bollen AW, Lamborn KR, Mohapatra G, Collins VP, Feuerstein BG (1999). Comparative genomic hybridization in patients with supratentorial and infratentorial primitive neuroectodermal tumors. Cancer.

[B4] Pomeroy SL, Tamayo P, Gaasenbeek M, Sturla LM, Angelo M, McLaughlin ME, Kim JY, Goumnerova LC, Black PM, Lau C, Allen JC, Zagzag D, Olson JM, Curran T, Wetmore C, Biegel JA, Poggio T, Mukherjee S, Rifkin R, Califano A, Stolovitzky G, Louis DN, Mesirov JP, Lander ES, Golub TR (2002). Prediction of central nervous system embryonal tumour outcome based on gene expression. Nature.

[B5] Saylors RL, Sidransky D, Friedman HS, Bigner SH, Bigner DD, Vogelstein B, Brodeur GM (1991). Infrequent p53 gene mutations in medulloblastomas. Cancer Res.

[B6] Badiali M, Iolascon A, Loda M, Scheithauer BW, Basso G, Trentini GP, Giangaspero F (1993). p53 gene mutations in medulloblastoma. Immunohistochemistry, gel shift analysis, and sequencing. Diagn Mol Pathol.

[B7] Adesina AM, Nalbantoglu J, Cavenee WK (1994). p53 gene mutation and mdm2 gene amplification are uncommon in medulloblastoma. Cancer Res.

[B8] Wang W, Kumar P, Whalley J, Schwarz M, Malone G, Haworth A, Kumar S (1998). The mutation status of PAX3 and p53 genes in medulloblastoma. Anticancer Res.

[B9] Frank AJ, Hernan R, Hollander A, Lindsey JC, Lusher ME, Fuller CE, Clifford SC, Gilbertson RJ (2004). The TP53-ARF tumor suppressor pathway is frequently disrupted in large/cell anaplastic medulloblastoma. Brain Res Mol Brain Res.

[B10] Giangaspero F, Rigobello L, Badiali M, Loda M, Andreini L, Basso G, Zorzi F, Montaldi A (1992). Large-cell medulloblastomas. A distinct variant with highly aggressive behavior. Am J Surg Pathol.

[B11] Eberhart CG, Kepner JL, Goldthwaite PT, Kun LE, Duffner PK, Friedman HS, Strother DR, Burger PC (2002). Histopathologic grading of medulloblastomas: a Pediatric Oncology Group study. Cancer.

[B12] McManamy CS, Lamont JM, Taylor RE, Cole M, Pearson AD, Clifford SC, Ellison DW (2003). Morphophenotypic variation predicts clinical behavior in childhood non-desmoplastic medulloblastomas. J Neuropathol Exp Neurol.

[B13] Jaros E, Lunec J, Perry RH, Kelly PJ, Pearson AD (1993). p53 protein overexpression identifies a group of central primitive neuroectodermal tumours with poor prognosis. Br J Cancer.

[B14] Burns AS, Jaros E, Cole M, Perry R, Pearson AJ, Lunec J (2002). The molecular pathology of p53 in primitive neuroectodermal tumours of the central nervous system. Br J Cancer.

[B15] Woodburn RT, Azzarelli B, Montebello JF, Goss IE (2001). Intense p53 staining is a valuable prognostic indicator for poor prognosis in medulloblastoma/central nervous system primitive neuroectodermal tumors. J Neurooncol.

[B16] Miralbell R, Tolnay M, Bieri S, Probst A, Sappino AP, Berchtold W, Pepper MS, Pizzolato G (1999). Pediatric medulloblastoma: prognostic value of p53, bcl-2, Mib-1, and microvessel density. J Neurooncol.

[B17] Neben K, Korshunov A, Benner A, Wrobel G, Hahn M, Kokocinski F, Golanov A, Joos S, Lichter P (2004). Microarray-based screening for molecular markers in medulloblastoma revealed STK15 as independent predictor for survival. Cancer Res.

[B18] Barel D, Avigad S, Mor C, Fogel M, Cohen IJ, Zaizov R (1998). A novel germ-line mutation in the noncoding region of the p53 gene in a Li-Fraumeni family. Cancer Genet Cytogenet.

[B19] Wetmore C, Eberhart DE, Curran T (2001). Loss of p53 but not ARF accelerates medulloblastoma in mice heterozygous for patched. Cancer Res.

[B20] Nagashima K, Yasui K, Kimura J, Washizu M, Yamaguchi K, Mori W (1984). Induction of brain tumors by a newly isolated JC virus (Tokyo-1 strain). Am J Pathol.

[B21] Marino S, Vooijs M, van Der Gulden H, Jonkers J, Berns A (2000). Induction of medulloblastomas in p53-null mutant mice by somatic inactivation of Rb in the external granular layer cells of the cerebellum. Genes Dev.

[B22] Zu Rhein GM (1983). Studies of JC virus-induced nervous system tumors in the Syrian hamster: a review. Prog Clin Biol Res.

[B23] Khalili K, Del Valle L, Otte J, Weaver M, Gordon J (2003). Human neurotropic polyomavirus, JCV, and its role in carcinogenesis. Oncogene.

[B24] Krynska B, Del Valle L, Croul S, Gordon J, Katsetos CD, Carbone M, Giordano A, Khalili K (1999). Detection of human neurotropic JC virus DNA sequence and expression of the viral oncogenic protein in pediatric medulloblastomas. Proc Natl Acad Sci U S A.

[B25] Del Valle L, Gordon J, Enam S, Delbue S, Croul S, Abraham S, Radhakrishnan S, Assimakopoulou M, Katsetos CD, Khalili K (2002). Expression of human neurotropic polyomavirus JCV late gene product agnoprotein in human medulloblastoma. J Natl Cancer Inst.

[B26] Fanning E, Knippers R (1992). Structure and function of simian virus 40 large tumor antigen. Annu Rev Biochem.

[B27] Lammie GA, Beckett A, Courtney R, Scaravilli F (1994). An immunohistochemical study of p53 and proliferating cell nuclear antigen expression in progressive multifocal leukoencephalopathy. Acta Neuropathol (Berl).

[B28] Ariza A, Mate JL, Fernandez-Vasalo A, Gomez-Plaza C, Perez-Piteira J, Pujol M, Navas-Palacios JJ (1994). p53 and proliferating cell nuclear antigen expression in JC virus-infected cells of progressive multifocal leukoencephalopathy. Hum Pathol.

[B29] Kononen J, Bubendorf L, Kallioniemi A, Barlund M, Schraml P, Leighton S, Torhorst J, Mihatsch MJ, Sauter G, Kallioniemi OP (1998). Tissue microarrays for high-throughput molecular profiling of tumor specimens. Nat Med.

[B30] Arthur RR, Dagostin S, Shah KV (1989). Detection of BK virus and JC virus in urine and brain tissue by the polymerase chain reaction. J Clin Microbiol.

[B31] Taoufik Y, Gasnault J, Karaterki A, Pierre Ferey M, Marchadier E, Goujard C, Lannuzel A, Delfraissy JF, Dussaix E (1998). Prognostic value of JC virus load in cerebrospinal fluid of patients with progressive multifocal leukoencephalopathy. J Infect Dis.

[B32] Yuan CC, Miley W, Waters D (2001). A quantification of human cells using an ERV-3 real time PCR assay. J Virol Methods.

[B33] Giordana MT, Duo D, Gasverde S, Trevisan E, Boghi A, Morra I, Pradotto L, Mauro A, Chio A (2002). MDM2 overexpression is associated with short survival in adults with medulloblastoma. Neuro-oncol.

[B34] Sarkar C, Pramanik P, Karak AK, Mukhopadhyay P, Sharma MC, Singh VP, Mehta VS (2002). Are childhood and adult medulloblastomas different? A comparative study of clinicopathological features, proliferation index and apoptotic index. J Neurooncol.

[B35] Maestro R, Dolcetti R, Gasparotto D, Doglioni C, Pelucchi S, Barzan L, Grandi E, Boiocchi M (1992). High frequency of p53 gene alterations associated with protein overexpression in human squamous cell carcinoma of the larynx. Oncogene.

[B36] Ho YS, Hsieh LL, Chen JS, Chang CN, Lee ST, Chiu LL, Chin TY, Cheng SC (1996). p53 gene mutation in cerebral primitive neuroectodermal tumor in Taiwan. Cancer Lett.

[B37] Kraus JA, Felsberg J, Tonn JC, Reifenberger G, Pietsch T (2002). Molecular genetic analysis of the TP53, PTEN, CDKN2A, EGFR, CDK4 and MDM2 tumour-associated genes in supratentorial primitive neuroectodermal tumours and glioblastomas of childhood. Neuropathol Appl Neurobiol.

[B38] Postovsky S, Ben Arush MW, Elhasid R, Davidson S, Leshanski L, Vlodavsky E, Guilburd JN, Amikam D (2003). A novel case of a CAT to AAT transversion in codon 179 of the p53 gene in a supratentorial primitive neuroectodermal tumor harbored by a young girl. Case report and review of the literature. Oncology.

[B39] Berrak SG, Ozek MM, Canpolat C, Dagcinar A, Sav A, El-Naggar A, Langford LA (2002). Association between DNA content and tumor suppressor gene expression and aggressiveness of atypical teratoid/rhabdoid tumors. Childs Nerv Syst.

[B40] Kinoshita Y, Shiratsuchi H, Tamiya S, Oshiro Y, Hachitanda Y, Oda Y, Suita S, Tsuneyoshi M (2001). Mutations of the p53 gene in malignant rhabdoid tumors of soft tissue and the kidney: immunohistochemical and DNA direct sequencing analysis. J Cancer Res Clin Oncol.

[B41] Ray A, Ho M, Ma J, Parkes RK, Mainprize TG, Ueda S, McLaughlin J, Bouffet E, Rutka JT, Hawkins CE (2004). A clinicobiological model predicting survival in medulloblastoma. Clin Cancer Res.

[B42] Padgett BL, Walker DL, ZuRhein GM, Varakis JN (1977). Differential neurooncogenicity of strains of JC virus, a human polyoma virus, in newborn Syrian hamsters. Cancer Res.

[B43] Matsuda M, Yasui K, Nagashima K, Mori W (1987). Origin of the medulloblastoma experimentally induced by human polyomavirus JC. J Natl Cancer Inst.

[B44] Krynska B, Otte J, Franks R, Khalili K, Croul S (1999). Human ubiquitous JCV(CY) T-antigen gene induces brain tumors in experimental animals. Oncogene.

[B45] Hayashi H, Endo S, Suzuki S, Tanaka S, Sawa H, Ozaki Y, Sawamura Y, Nagashima K (2001). JC virus large T protein transforms rodent cells but is not involved in human medulloblastoma. Neuropathology.

[B46] Kim JY, Koralnik IJ, LeFave M, Segal RA, Pfister LA, Pomeroy SL (2002). Medulloblastomas and primitive neuroectodermal tumors rarely contain polyomavirus DNA sequences. Neuro-oncol.

[B47] Rollison DE, Utaipat U, Ryschkewitsch C, Hou J, Goldthwaite P, Daniel R, Helzlsouer KJ, Burger PC, Shah KV, Major EO (2005). Investigation of human brain tumors for the presence of polyomavirus genome sequences by two independent laboratories. Int J Cancer.

[B48] Weggen S, Bayer TA, von Deimling A, Reifenberger G, von Schweinitz D, Wiestler OD, Pietsch T (2000). Low frequency of SV40, JC and BK polyomavirus sequences in human medulloblastomas, meningiomas and ependymomas. Brain Pathol.

[B49] Lopez-Rios F, Illei PB, Rusch V, Ladanyi M (2004). Evidence against a role for SV40 infection in human mesotheliomas and high risk of false-positive PCR results owing to presence of SV40 sequences in common laboratory plasmids. Lancet.

